# Risk Assessment of Fishing Trawl Activities to Subsea Pipelines of Sabah and Labuan Waters

**DOI:** 10.1155/2020/6957171

**Published:** 2020-07-23

**Authors:** Ahmad Faizal Ahmad Fuad, Mohd Hafizi Said, Khalid Samo, Mohd Asamudin A. Rahman, Mohd Hairil Mohd, Ismail Zainol

**Affiliations:** ^1^Nautical Science Program, Faculty of Maritime Studies, Universiti Malaysia Terengganu, Kuala Nerus 21030, Terengganu, Malaysia; ^2^Maritime Technology Program, Faculty of Ocean Engineering Technology and Informatics, Universiti Malaysia Terengganu, Kuala Nerus 21030, Terengganu, Malaysia; ^3^Universiti Kuala Lumpur, Institute of Marine Engineering Technology, Dataran Industri Teknologi Kejuruteraan Marin, Bandar Teknologi Maritim, Jalan Pantai Remis, Lumut 32200, Perak, Malaysia

## Abstract

*Introduction*. Trawling is a method of catching fish in a large volume where fish nets are pulled through water using one or two boats. Bottom trawling is where the nets are pulled over on the seabed. The gear of the bottom trawling would impact the exposed subsea pipeline, on the seabed. Subsea pipelines transport crude oil and gas from the offshore platform to shore facility. This study assesses the risk of fish trawling activities to the subsea pipelines at Sabah and Labuan offshore. The specification of trawl equipment used by local trawlers in Sabah was determined by the on-site survey. The frequency of a fish trawler crossing over the pipelines was calculated based interview on operation and site survey. The calculation of the pull-over load of the otter board was calculated using the DNVGL algorithm. The severity and frequency index of the risk matrix was developed based on literature review. Results showed that the pull-over load of the otter board would not damage the pipelines. The risk posed by the fish trawler activity to the pipelines is low and moderate.

## 1. Introduction

**Figure 1:**
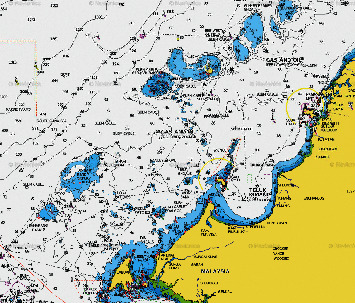
Oil fields and subsea pipelines at Sabah West Basin (Source: Navionics [2]).

Sabah is known for its fresh sea catches. Seas of Sulu, Sulawesi, and South China, which surround Sabah, contribute to 41.8% in marine fish catches in Malaysia. Sabah fisheries and aquaculture industries produce nearly 200,000 metric tons of fish annually and contribute to Sabah's annual Gross Domestic Product by 2.8%. The marine capture fishery is the major contributor, which accounts for about 80% of the statistics. This is a catalyst by various fishing gear methods used by the fisherman in Sabah. The main fishing method that contributes to the total catches is the trawl net. Trawling is a method of fishing where fish nets are pulled through water using one or two boats. Trawl can be split into bottom trawling and midwater trawling [1]. Apart from fisheries and aquaculture industries, Sabah has an oil and gas industries with a reservoir that consist of West Sabah Basin, Northwest Sabah Basin, and Northeast Sabah deep-water area. Most oil and gas come from the West Basin, namely, Erb West, Tembungo, and Kababangan oil fields as shown in Figure 1.

However, the existing subsea pipelines connecting oil field to shore are affected by fishing activities especially the bottom-trawling. The interaction may cause damage to the subsea pipelines, which eventually leak and cause marine pollution. This interaction is considered as a third-party impact due to the human activity at sea. The third-party impact is the impact caused by external activities such as trawling, anchoring, and dropped objects [3].

A number of studies have been conducted on the interaction between trawl gear and subsea pipelines [4–8]. DNV had introduced the algorithm to calculate the pull-over load and hooking of trawl gear on the subsea pipelines [9]. The behaviour of X65 pipeline during impact has been studied by Kristoffersen et al. [10]. Longva et al. [11] had conducted a load test of fishing gear on subsea pipelines. The study had identified the friction of board pipe, the tension of wire between the board and trawl net, the towing line drag properties, and the direction of over-trawling that profoundly influenced the impact's load. Wu et al. [12] had proposed a new approach to quantify the probability of the trawl board hooking by using simulation tools and statistical data. DNV had updated their recommended practice on interference between trawl gear and pipelines in 2017 [9].

Hitherto, no study has been conducted on the fish trawlers activities to subsea pipelines, particularly at Sabah offshore. Therefore, the objective of this research is to determine the impact of the pull-over load of trawlers to the subsea pipelines from Sabah and Labuan offshore to shore gas and crude terminal, respectively.

## 2. Methods

**Figure 2:**
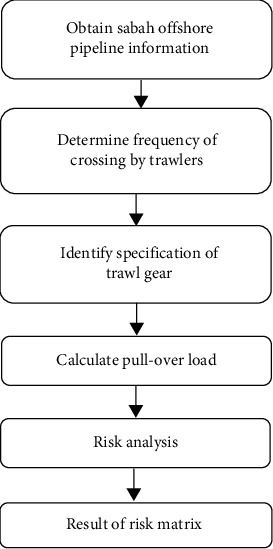
Overall research activities.

In this research, several methods were used to achieve the main objective. The overall research activities are shown in Figure 2. The explanation of each process is given in the following paragraphs.

The first step of the research is to obtain information on oil and gas pipelines from offshore fields to shore facilities at Sabah and Labuan. The data were obtained from the relevant MAL chart series, produced by the Malaysia National Hydrographic Centre. The electronic navigation chart series are available from C-Map and Navionics. The pipelines data were also obtained from the oil and gas company that owns the pipelines.

The second step is to determine the frequency of crossing by trawlers on the pipelines. According to the Sabah Fisheries Department, less than 30% of fishing vessels in Sabah is fitted with the Automatic Identification System (AIS). Thus, the AIS data for trawlers were not available. To determine the number of crossings on pipelines, an alternative way was developed.

The first method of the second step was to determine the typical operation of trawlers in Sabah. The information was gathered by conducting a survey on the three operator fish trawlers at Kota Kinabalu fish jetty. The information was the location of fishing ground, speed and duration to the fishing ground, trawling speed and duration, the number of trawling per day, duration of fishing, speed and duration to return to fish landing jetty, duration of transfer cargo to jetty, and replenishment before going back to fishing. The second method of the second step is to do a site verification survey by boating along the route of the pipelines. The course, speed, name, and type of fishing vessels detected on-site were recorded. The third method of the second step is to determine the density of fishing trawler per area of fishing ground. This step was started by identifying fishing grounds in Sabah waters. The area for each fishing ground was marked and measured by using the Google Earth application.

The third step was to identify the specification of the trawl gear and otter board used by trawlers. The site survey was conducted to fishing trawlers at the Kota Kinabalu fishing port and shipyard at Sepanggar Bay. During the survey, the dimension of the otter board was measured, and the thickness of the warp line was measured.

The fourth step was to calculate the force of the pull-over load by using equations (1)–(4) developed by DNV [9]. The pull-over load is the horizontal and vertical forces from the trawl boards acting towards the subsea pipeline. It shall be applied as single point load to the pipeline under consideration [13].

The equation for the pull-over load of an otter board or trawl door is given below [9]:

(1) $$\begin{array}{c} F_{P}=C_{F}.V{\left(m_{t}k_{w}\right)}^{1/2} \end{array}$$

where *F*_*P*_ is the pull-over load of an otter board/trawl door, *k*_*w*_ is the warp line stiffness, *V* is the trawling velocity, *m*_*t*_ is the steel mass of board/beam with shoes, and *C*_*F*_ is an empirical coefficient.

*C*_*F*_ is determined by

(2) $$\begin{array}{c} C_{F}=8.0\cdot \left(1-e^{-0.8\bar{H}}\right), \end{array}$$

where $\bar{H}$ is a dimensionless height and is determined by

(3) $$\begin{array}{c} \bar{H}=\frac{H_{\text{sp}}+OD/2+0.2}{B}, \end{array}$$

where *H*_sp_ is the span height (negatively for the partly buried trenched pipeline). OD is the pipeline outer diameter including coating_._*B* is half of the trawl board height.

The warp stiffness, *k*_*w*_, is assumed as

(4) $$\begin{array}{c} k_{w}=\frac{3.5.10^{7}}{L_{W}}, \end{array}$$

where *L*_*W*_ is the length of warp line in meter.

The fifth step was to conduct the risk analysis, which consists of the frequency index, severity index, and risk matrix. The frequency index and severity index will be developed based on DNV GL and ISO publications.

The frequency index developed in this study is shown in Table 1. The table is a 9-point scale ranging from 0 to 8,100 frequencies with an increment of 900 between categories. The table is developed based on the DNV GL report on recommended failure rates for pipelines by adapting table criteria for score assessment, threats related to shipping loss, emergency anchoring, and dragged anchors from anchored ships [14]. The table has 3 scores as follows: 0, 1, and 2 for the number of crossing less than 90,000, between 90,000 and 180,000, and above 180,000, respectively. The adaptation was by taking 1% of the scores of each category from the DNV GL table and developed the ratings in 9 scales. This adaptation would increase the impact probability to 100 per cent, which is due to the impact of trawl gear on the pipeline on each crossing.

The severity index in Table 2 was developed based on research conducted by Kristoffersen et al. [10]. However, no results of the calculated force are shown in the table in this section. The test conducted in the research is in accordance with DNV-RP-F111: interference between trawl gear and pipelines [9]. Results from the research were used to determine the magnitude of the force that resulted in the extent of damage to the pipeline, which also subjects to the thickness of the pipeline. A pipeline's load resistance against external interference primarily depends on the pipeline diameter and wall thickness. Tests have shown that the most commonly used excavators and construction equipment do not exercise enough load to cause leaks or rupture to pipelines with a wall thickness larger than 11-12 mm [14]. The index number and meaning of the index of severity in Table 2 is developed based on ISO 17776 Risk Matrix with index number starting from 0 to 4 [15].

Table 3 is the Risk Matrix Table for fishing activities on pipeline, which is used to determine the result of the risk index. Table 3 combined the severity index and frequency index in one table and was used to calculate the summation between the frequency and severity index for a particular pipeline. The result of the summation and the corresponding level of risk are shown in Table 4. There are five levels of risk matrix shown in Table 4 from very low to very high risk.

## 3. Results and Discussion

**Figure 3:**
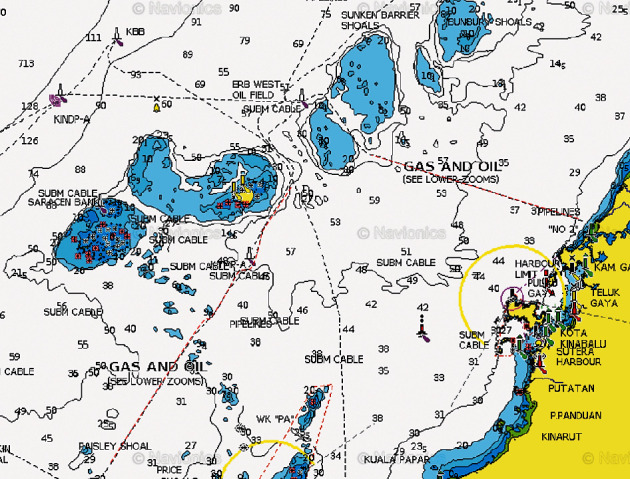
Pipeline ID 85 Erb West to SBGAST (red dotted line right) and ID80 Erb West to LGAST (red dotted line left). Coordinates pipeline ID85 from sea to shore is 6° 15.126′N 115° 50.156′E and 6° 9.730′N 116° 7.189′E, respectively.

**Figure 4:**
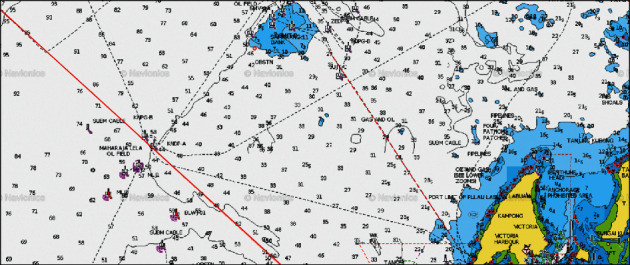
Pipeline ID107 and ID144A Route Semarang to Labuan Gas Terminal (LGAST) (red line). Coordinates pipeline ID144A from sea to shore is 5° 33.00′N 114° 57.922′E and 5°16.584′ N 115° 8.730′E, respectively.

There were four pipelines that were identified in the study. Pipelines ID85 and ID80 cross from Erb West oil field to Sabah Gas Terminal Kota Kinabalu (SBGAST) and to Labuan Gas Terminal (LGAST), respectively (Table 5). Pipelines ID107 and 144A from Semarang oil field to LGAST are shown in Figures 3 and 4.

### 3.1. The Density of Fishing Trawler in Sabah Waters

**Figure 5:**
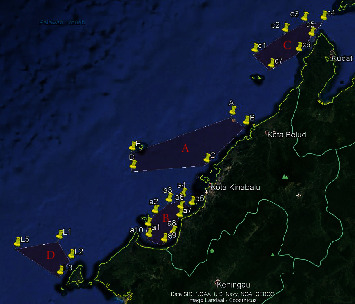
Fishing ground in west coast Sabah and Labuan. The coordinates for area B are a4: 5°52′10.62″N 115°53′9.66″E; a5: 5°49′5.10″N 115°56′58.14″E; a9: 5°33′40.32″N 115°46′52.44″E; and a10: 5°35′18.85″N 115°40′39.49″E. Coordinates for area C are c1: 6°51′28.20″N 116°17′59.82″E; c3: 7° 5′24.37″N 116°37′8.46″E; c5: 6°59′34.08″N 116°40′22.26″E; and c7: 6°45′51.84″N 116°25′4.38″E.

According to Kristoffersen et al. [10], there are four main fishing grounds of Sabah, as shown in Figure 5. Fishing ground A is located between Erb West oil field and Kota Kinabalu mainland. Fishing ground B is located at Teluk Kimanis. Fishing ground C is located between Mantani Island and the mainland, and fishing ground D is located between Semarang oil field and Labuan mainland.

**Figure 6:**
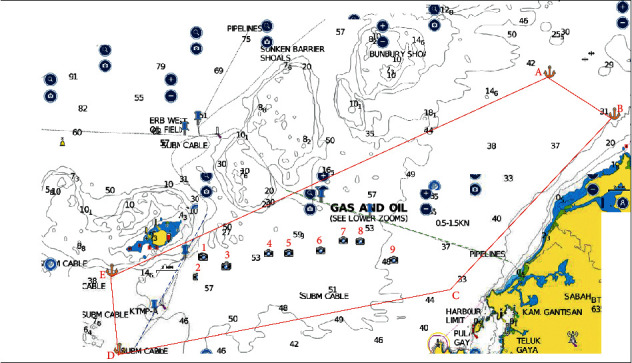
Trawling area that involves Erb West to Labuan Gas Terminal pipeline (blue dotted line) and Erb West to Sabah Gas Terminal pipeline (green dotted line). The positions of the trawling area as follows: A: 6° 26.17′ N 116° 10.76′ E; B: 6° 22.50′ N 116° 16.53′ E; C: 6° 6.55′ N 116° 1.69′ E; D: 6° 1.98′ N 115° 32.44′ E; and E: 6° 9.21′ N 115° 31.75′ E. The area is 488 nm square. The camera icon depicts the position of 10 trawlers. The nature of seabed in this area is mud.

The area of fishing ground A is 488 nm^2^. Results of site survey at ground A show that twelve trawlers were found, as shown in Figure 6 (the positions depicted by camera icon). The density of fishing trawlers in an area was obtained by dividing the area measured with the number of fishing trawlers found; thus, the density of fishing trawler in area A is 40.7 nm^2^ per trawl. Fishing ground A is used as the benchmark for density of trawler because it has the highest number of fishing trawlers registered at its coast (Kota Kinabalu) compared to other fishing ground, and it is also the biggest fishing ground in Sabah. By using the result from ground A, the density of trawler for fishing ground B, C, and D is calculated. The area for ground B, C, and D is 157 nm^2^, 213 nm^2^, and 156 nm^2^, respectively. The density of trawler for ground B, C, and D is 4, 5, and 4, respectively.

### 3.2. Typical Operation of Fishing Trawlers in Sabah Waters and Frequency of Crossing

An interview with three skippers of fishing trawler had identified the typical operation of the fishing trawlers in Sabah waters. The information of the operation is applied to fishing ground A, an area between Erb West oil field and the coast of Kota Kinabalu to Karambunai Sabah (Figure 4). The result for fishing ground A is as follows:

- The duration of one cycle of fishing trawl operation is 10 days, which consists of 0.5 days going to a fishing ground, 7 days of trawling, 0.5 days to return to fish landing jetty, and 2 days for discharge cargo and replenishment.
- The trawling operation is conducted 5 times per day, where the duration for each trawling operation is 4 hours, namely 1 hour to deploy and retrieve net, and 3 hours to tow net.
- The trawl distance per day is 45 nm, which resulted from 5 times trawl per day, multiply 3 hours duration per trawl, and multiply speed 3 knots.
- The distance of the fishing area is 28 nm, which is measured from pipeline Erb West–Labuan Gas Terminal to pipeline Erb West–Sabah Gas Terminal in Figure 2. For a trawler that makes a U-turn after 28 nm trawling to make a new trawl leg, with a trawling distance of 45 nm, a vessel would cross 1 pipeline twice and 1 pipeline once. However, by taking into consideration the slight increase of speed, lesser time in deployment, and retrieval of trawl gear, it is assumed that a trawler may cross these two pipelines 2 times per day.
- Monthly crossing: for 3 fishing trips at 7 days actual trawling operation, the trawling days is 21 days. Each trawler would cross 42 times on each pipeline (21 days trawling per month *x* 2 times crossing per day).
- Annual crossing: assuming 11 months annual operation (one month reserved for maintenance and repair), each trawler would cross one pipeline 462 times (42 times cross/month *x* 11 months)
- The density of fishing trawler for the area is 12. Therefore, the number of annual crossing is estimated at about 5544 crossings (i.e., 12 trawlers *x* 462 crossed per vessel) for each pipeline in area A.

**Figure 7:**
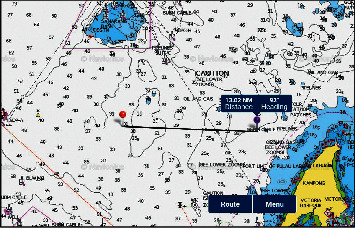
The width distance of area D (Labuan) fishing ground. The nature seabed in this area is sand and mud. The coordinates for area Labuan D are as follows: L1: 5°32′4.74″N 115° 4′54.54″E; L2: 5°24′37.98″N 115°10′2.76″E; L3: 5°17′45.18″N 115° 6′9.96″E; and L5: 5°27′52.68″N 114°47′59.22″E (Figure 5).

The results of fishing ground D are as follows:

- 10 days per cycle of operation (0.5 days to fishing grounds, 7 days trawling, 0.5 back to port, and 2 days to land fish and replenish fuel and supply).
- Trawler density: 40.7 nm^2^ area density per trawler at area A is applied to other areas in Sabah and Labuan. Therefore, for area D with 156 nm^2^, the density of trawlers for the area at one time is 4 trawlers (156 nm^2^/40.7 nm^2^).
- Trawling distance of a trawler per day is 45 nm (5 times trawl per day *x* 3-hour trawl *x* 3 knots).
- Length of fishing area = 13 nm (From Semarang–Labuan LGAST ID: 107 and ID: 144) (Figures 4 and 5).
- A trawler would make a U-turn after a distance of 13 nm for new trawl leg (Figure 7).
- With a distance of 45 nm trawl distance, a vessel would cross 2 pipelines (ID: 107 and ID: 144) 4 times crossing per day (Figure 5).
- Monthly crossings: 84 crossings/trawler/pipeline (at three 7-day fishing trips/month), i.e., 7 days *x* 3 trips *x* 4 times crossing per day.
- Annual crossings: 924 crossings/trawler/year (assuming 11 months operation, less than 1 month for maintenance and repair), i.e., 84 crossings *x* 11 months.
- Based on the density of fishing trawler for the area which is 4 (see above assumption), the total number of annual crossing = 3,696 (4 trawlers *x* 924 crossings).

The frequency index table for the number of crossing on a pipeline by trawlers in a year is developed based on the assumed frequency of trawlers passing on the pipelines. This frequency is developed based on the assumption of the vessel speed, route taken, distance travelled, and commonly practiced by the fishermen. The frequency developed had to be developed based on the assumption because accurate information leading to the frequency was not available.

### 3.3. Specification of Trawl Gear

**Figure 8:**
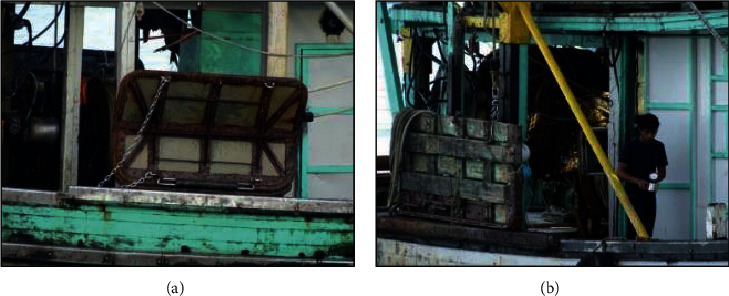
Steel vee door (a) and common flat wooden door (b) used by trawlers in Sabah.

The type of trawl gear used in Sabah is the typical otter trawl gear, which is using the polyvalent or rectangular board. This type of trawl gear consists of a pair of otter boards, warp line, and net. There are two types of otter board used by trawlers in Sabah, namely the steel otter board and the steel-reinforced wooden otter board as shown in Figure 8.

The types and dimension of the otter board are shown in Table 6. The category of fish trawlers that are using these otter boards must have sufficient power to tow the heavy fishing gear at 3 knots. Therefore, the engine horsepower is 350 HP and above. The majority of engine used in the fishing trawlers have horsepower between 350 hp to 500 hp. The diameter of the warp line used is 2.5 cm.

### 3.4. Calculation of the Pull-Over Load

The calculation of the pull-over load was done using equations (1)–(4). The inputs to the equations are trawl depth of 60 meters (Figure 2), trawling speed of 3 knots (1.54 m/s), 300 kg steel vee door specification (Tables 6 and 7), and span height on the seabed. All the pipelines are buried. However, by taking into consideration that the pipelines sit on the seabed, the span height is zero [16].

Based on the calculation, the result of the pull-over load of a trawl board on Erb West to Labuan Gas Terminal is 24.01 kN and on Erb West to Sabah Gas Terminal is 25.98 kN (Table 8). These results were compared with the yield strength of the pipelines in Table 7. These results show that the trawl board's pull-over load on Erb West to Labuan Gas Terminal and Erb West to Sabah Gas Terminal pipelines is far lower than the yield strength of both pipelines. The percentage of the pull-over load over yield strength of Erb West to Labuan Gas Terminal and Erb West to Sabah Gas Terminal pipelines are 7.8% and 4.5%, respectively. Therefore, the force of the pull-over load of the trawl boards would not give a significant impact to damage the pipelines. However, due to age factor and corrosion, the yield strength of the pipelines should be lower than new condition [14].

### 3.5. Calculation of the Frequency Index, Severity Index, and Risk Matrix for Fishing Activities

Both severity index for pipeline Erb West to the Labuan Gas Terminal and Erb West to the Sabah Gas Terminal is zero because the impact force from the otter board resulted in no damage to the pipelines. The risk matrix results for both pipelines depend on the frequency of crossing as shown in Table 9.

Based on the observation of fishing trawl operating in Sabah (Area A) and Labuan (Area D), the fish trawl skippers are aware that they were operating within the area with offshore pipelines but do not know the exact location of the pipelines. The reason that they fish in the area is that the yield from the catch was good, and the seabed is flat and suitable for trawl operation. According to Rouse et al. [17], snagging may damage fishing gear, disrupt the fishing operation, and may cause injuries to crew. However, no fish trawlers in the study area (Sabah and Labuan) had snagged their fishing gear with the pipelines. This may be due to the pipelines buried in the muddy seabed. Therefore, the fishermen did not consider the pipelines as a snagging hazard.

## 4. Conclusion

The trawling method used in Sabah waters is the bottom otter trawl. This type of trawling would cause contact between the trawl boards with subsea pipelines. Based on the specification of the trawl board and the subsea pipeline of the study area, the impact force of the trawl board's pull-over load is lower that the force would cause slight damage to the pipelines. The interaction between the otter boards and subsea pipelines in West Sabah area and Labuan waters would not damage the subsea pipelines. However, the frequency of the crossing in Labuan and Sabah (Kota Kinabalu) waters are moderate and high, respectively. Based on the severity index and frequency index, the risk of fishing activities Labuan and Sabah (Kota Kinabalu) waters are low risk and moderate risk, respectively.

## Figures and Tables

**Table 1:** Frequency index of fishing vessel crossing.

Index	Meaning	Range
0	Extremely low	0–900
1	Very low	901–1800
2	Low	1801–2700
3	Slightly low	2701–3600
4	Moderate	3601–4500
5	Slightly high	4501–5400
6	High	5401–6300
7	Very high	6301–7200
8	Extremely high	7201–8100

**Table 2:** Severity index of the pull-over force/load.

Index	Meaning	Calculated force (*F*_p_)
0	No damage	No data
1	Slight damage	No data
2	Minor damage	No data
3	Local damage	No data
4	Major damage	No data

**Table 3:** Risk matrix table for fishing activities on pipeline.

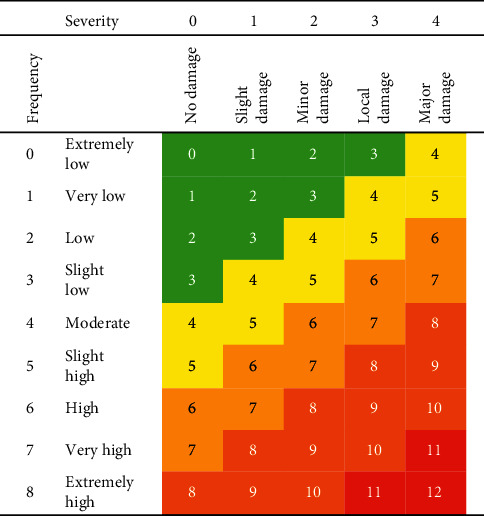

**Table 4:** Risk matrix table of fishing activities impact on pipeline.

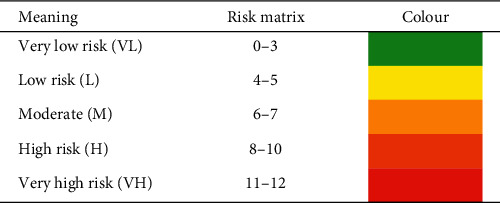

**Table 5:** Pipelines information.

Pipeline name	Pipe length (km)	Pipe diameter (inch/cm)	Design code	WT (mm)	Yield strength kN/m^2^
ID85 Erb West to SBGST	60.4	16/40.6	API 5L X60	15.9	4.2 × 10^5^ @ 60 ksi
ID80 Erb West to LGAST	140.4	14/35.5	API 5L X42	9.53	2.9 × 10^5^ @ 42 ksi
ID107 Semarang to LGAST	46	14/35.5	API 5L X52	9.53	3.55 × 10^5^ @ 52 ksi
ID144A Semarang to LGAST	47.71	20/50.8	API 5L X65	11.1	4.5 × 10^5^ @ 65 ksi

**Table 6:** Types of fish trawl otter board used in Sabah and Labuan.

Trawler categories	Types of otter boards	Dimensions in cm (L × W × T)	Material used	Weight (kg) of one otter board
350 HP and above	Steel vee door	190 × 106 × 6.5	Steel	300
350 HP and above	Common flat wooden door	230 × 115 × 5	Steel frame and wood	250
350 HP and above	Steel vee door	165 × 110 × 5	Steel	210

**Table 7:** Subsea pipeline Erb West oil field to Labuan data.

Pipeline name	Pipe length (km)	Pipe diameter (inch/cm)	Design code	WT (mm)	Yield strength (kN/m^2^)	Pull-over load (kN)
ID80 Erb West to LGAST	140.4	14/35.5	API 5L X42	9.53	2.9 × 10^5^ @ 42 ksi	24.01
ID85 Erb West to SBGST	60.4	16/40.6	API 5L X60	15.9	4.2 × 10^5^ @ 60 ksi	25.98
ID107 Semarang to LGAST	46	14/35.5	API 5L X52	9.53	3.55 × 10^5^ @ 52 ksi	28.19
ID144A Semarang to LGAST	47.71	20/50.8	API 5L X65	11.1	4.5 × 10^5^ @ 65 ksi	38.183

**Table 8:** Level of damage according to force of impact.

Pipeline name	Thickness (mm)	Extent of damage	Force (kN)	Yield strength (kN/m^2^)	Pull-over load *F*_p_ (kN)
ID80 Erb West/LGAST	9.53	Major	172.011	2.9 × 10^5^ at 42 ksi	24.01
		Local	127.067		
		Minor	72.946		
		Slight	47.062		
ID85 Erb West/SBGST	15.9	Major	286.985	4.2 × 10^5^ at 60 ksi	25.98
		Local	212.000		
		Minor	121.704		
		Slight	78.519		
ID107 Semarang/LGAST	9.53	Major	172.011	3.55 × 10^5^ at 52 ksi	28.19
		Local	127.067		
		Minor	72.946		
		Slight	47.062		
ID144A Semarang/LGAST	11.1	Major	200.348	4.5 × 10^5^ at 65 ksi	38.183
		Local	148.000		
		Minor	84.963		
		Slight	54.815		

**Table 9:** Calculated risk matrix.

ID &name	Frequency crossing	Frequency index	Pull-over load (kN)	Severity index	Risk matrix
ID80 Erb West/LGAST	3696	4	24.01	0	4 (low risk)
ID85 Erb West/SBGST	5544	6	25.98	0	6 (moderate)
ID107 Semarang/LGAST	3696	4	28.19	0	4 (low risk)
ID144A Semarang/LGAST	3696	4	38.183	0	4 (low risk)

## Data Availability

The data used to support the findings of this study are available from the corresponding author upon request.
