# Corrigendum to “Risk Assessment of Fishing Trawl Activities to Subsea Pipelines of Sabah and Labuan Waters”

**DOI:** 10.1155/2020/7014928

**Published:** 2020-12-03

**Authors:** Ahmad Faizal Ahmad Fuad, Mohd Hafizi Said, Khalid Samo, Mohd Asamudin A. Rahman, Mohd Hairil Mohd, Ismail Zainol

**Affiliations:** ^1^Nautical Science Program, Faculty of Maritime Studies, Universiti Malaysia Terengganu, Kuala Nerus 21030, Terengganu, Malaysia; ^2^Maritime Technology Program, Faculty of Ocean Engineering Technology and Informatics, Universiti Malaysia Terengganu, Kuala Nerus 21030, Terengganu, Malaysia; ^3^Universiti Kuala Lumpur, Institute of Marine Engineering Technology, Dataran Industri Teknologi Kejuruteraan Marin, Bandar Teknologi Maritim, Jalan Pantai Remis, Lumut 32200, Perak, Malaysia

In the article titled “Risk Assessment of Fishing Trawl Activities to Subsea Pipelines of Sabah and Labuan Waters” [1], some information was omitted in the Acknowledgments section. The corrected section is as follows.
